# Research on the Relationship between Exposure to Dioxins and Cancer Incidence in Vietnam

**DOI:** 10.3390/toxics10070384

**Published:** 2022-07-11

**Authors:** Tuong Phi Vuong

**Affiliations:** Investigation Center No 69, Hanoi 118700, Vietnam; tphivuong@yahoo.com

**Keywords:** dioxins, cancer, incidence, risk ratio, Vietnam

## Abstract

The aim of this literature review is to discover whether there is a relationship between exposure to dioxins and cancer incidence in the hotspot regions of Vietnam by estimating the risk ratio index. The results of the study show that the incidence of cancer (soft tissue sarcoma; Hodgkin’s and non-Hodgkin’s lymphoma; lung, prostate, and liver cancer) in the dioxin-exposed Vietnamese population is much higher than the results of studies published in other countries because of the high levels of dioxins in South Vietnam, where Agent Orange was sprayed during the war. Further studies on the health effects of dioxins in the Vietnamese population, including cancer incidence, should be conducted with improved research methods.

## 1. Introduction

Dioxin is a general term that describes a group of hundreds of toxic organochlorine compounds that are highly persistent in the environment. The most toxic compound is 2,3,7,8-tetrachlorodibenzo-p-dioxin or TCDD. This dioxin compound was classified by the International Organization for Research on Cancer (IARC) as a human carcinogen (Group 1) based on animal studies, novel knowledge about its cytotoxicity mechanisms [1], and human studies on occupational exposure [2].

Dioxins have caused ecological disasters in Seveso (Italy) [3], Times Beach (Missouri), and the Love Canal (New York), but the most severe and long-lasting contamination occurred in South Vietnam, where Agent Orange was sprayed by the US for 10 years [4].

There are numerous epidemiological studies concerning the relationship between dioxins and cancer development in humans using different statistical indexes such as the odds ratio (OR), risk ratio (RR), standard incidence ratio (SIR), and standardized mortality ratio (SMR) for the cancer incidence or mortality of populations exposed to dioxins. However, epidemiological data on the carcinogenic potential of dioxins in Vietnamese people still have certain limitations because they have only been reported in observational epidemiological studies. Thus, the aim of this literature review is to provide a quantitative assessment of the association from an epidemiological point of view by estimating the risk ratio (RR) of cancer incidence in Vietnamese people exposed to dioxins.

## 2. Materials and Methods

### 2.1. Data Sources, Search Strategy and Selection Criteria

Literature searches were conducted in Google Scholar in both English and Vietnamese to identify eligible studies. The following terms were used in the search procedure: (“dioxin” or “TCDD” or “Agent Orange) AND (“cancer incidence”) AND (“Vietnamese” or “Vietnam”).

Studies were eligible for inclusion if all of the following criteria were fulfilled: (1) the studies evaluated the association between dioxin/TCDD and cancer incidence; (2) the risk ratio (RR) or odds ratio (OR) estimates were available for evaluation; (3) the articles as full papers were written in English or Vietnamese. (4) Reviews, meeting abstracts, notes, comments, editorials, and case reports were excluded because of their limited data.

### 2.2. Data Extraction and Synthesis

For the English literature review, we concentrated on published studies conducting a meta-analysis and official literature concerned with dioxins and cancer. The following information was extracted from each study: author(s), year of publication, country of study, study period, population characteristics (sample size, gender and age), and cancer subtypes risk ratios.

The Vietnamese literature review was followed by a data analysis to assess the association between dioxin exposure and all cancer incidences. For studies covering the relationship between exposure to dioxins and human cancer development, the risk ratio was estimated from the cumulative incidence:

Risk Ratio = CI_e_/CI_u_

where CI_e_ is the cumulative incidence in the ‘exposed’ group, and CI_u_ is the cumulative incidence in the ‘unexposed’ group. A 95% confidence interval (95% CI) was calculated.

## 3. Results

### 3.1. Dioxins and Cancer around the World

The latest publication from the IARC was published in 2012 [2] and comprehensively reviews epidemiological studies for any relationships between dioxins and human cancer development. Many studies have been carried out in the US [5,6,7,8,9], Germany [10,11], and Italy [12], and there have also been some multinational studies [13], including many cohort and case–control studies, on very large numbers of participants exposed to dioxins, mainly due to occupational exposure or environmental pollution incidents.

These studies were then analyzed by Xu et al. in 2016 in a systemic review [14], which involved 18,969 cancer cases and 3,155,159 participants included in 5 cohort studies and 5 case–control studies to assess the association between external exposure to dioxins and cancer incidence. Six of the studies with a cancer incidence RR are described in more detail in Table 1.

The pooled RR of the all-cancer incidence with external exposure to TCDD was 1.01 (95% CI: 0.97–1.06), indicating no significant association between external exposure to TCDD and cancer incidence. There was significant heterogeneity across the included studies (I2 = 73.5%, *p* < 0.001). A subgroup analysis was conducted according to cancer subtypes (Table 2).

All of the pooled RRs of exposure incidence cancer subtypes were not significant, including those for Hodgkin’s lymphoma, non-Hodgkin’s lymphoma, and soft tissue sarcoma (Table 2). Other cancer subtypes were also studied, but there was no information about their exposure incidence risk ratios.

One of the most important results of the study by Xu et al. [14] was the evidence of a significant positive association between cancer incidence, not only with external exposure, but also with blood TCDD level (the pooled RR for all cancer incidence was 1.57 (95% CI: 1.21–2.04)) (data not shown in Table 1). The subgroup analysis of blood incidence was not conducted due to the limited data.

Based on the literature review of prior studies such as Veterans and Agent Orange: Update 11 (2018) [17] and different annual Agent Orange and dioxin studies, committee updates from the Vietnam Veterans of America determined that no other changes in the association level between the relevant exposure levels and other cancer types are currently observed, as there are either no published studies, or new evidence has supported the findings of earlier updates. Thus, the current findings on cancer can be summarized as follows: there is sufficient evidence of an association between dioxins and soft tissue sarcomas, Hodgkin’s lymphoma, and non-Hodgkin’s lymphoma; there is limited or suggestive evidence of an association between dioxins and bladder cancer, laryngeal cancer, cancers of the lung, bronchus, and trachea, prostate cancer, multiple myeloma, and AL amyloidosis; there is inadequate or insufficient evidence to determine whether there is an association between dioxins and any other specific type of cancer.

In the 2020 issue of the annual Agent Orange Newsletter [18], the US Department of Veterans Affairs presumed that prostate cancer, respiratory cancers, chronic B-cell leukemias, and multiple myeloma are related to herbicide exposure, along with soft tissue sarcomas and Hodgkin’s and non-Hodgkin’s lymphomas. However, more clear evidence is needed to further epidemiological studies.

### 3.2. Dioxins and Cancer in Vietnam

Research by Kahn (1988) [19], Schecter (1990) [20], and Michalek (1990) [7] shows that the concentration of dioxins stored in the adipose tissue in the bodies of the US veterans who participated in the Vietnam War was 600 times higher than normal (600 ppt vs. 1–2 ppt) after many years. The TCDD geographic distribution study showed that TCDD rates were higher in the Vietnamese population in the southern and central provinces [21]. TCDD concentrations ranged from non-detectable to 1648 pg/g of lipids in human biological samples obtained from 1970 to 2017, while that of US adults was only 5.2 pg/g (measured by the US National Health and Nutrition Examination Survey in 2003–2004). The highest dioxin concentration recorded was in a fisherman at the lake in the Da Nang Air Base, which was over 1000 ppt [22]. In addition to an increase in the dioxin levels in the bodies of the Vietnamese people, dioxin levels also increased in the food and wildlife in these contaminated areas. Dioxin has been found in Vietnamese soil or sediments at concentrations of up to 1 million ppt 30–40 years after the areas had been sprayed with Agent Orange. The lipid-adjusted dioxin toxic equivalent (TEQ) in poultry in Vietnam was 25.8 pg/g compared to 0.018 pg/g for the US samples (Lorber et al., 2009) [23]. The mean TCDD concentration reported in eggs from Vietnam was approximately 4 pg/g, an order of magnitude higher than that from the US (in California) in the late 1990s (Goldman et al., 2000) [24].

Research on diseases related to toxic chemicals/dioxins in Vietnam veterans by Le et al. [25] was conducted in the period between 1994–2004, in which the patient files of and structured questionnaires administered to 47,893 veterans in eight provinces and cities across the country who had participated in the Vietnam War, which took place from 1962 to April 1975, were analyzed. The cancer incidence in the group with a history of exposure to toxic chemicals/dioxins was statistically significantly higher than in the unexposed group (*p* < 0.01). The incidence of lung cancer was 1.30%; Hodgkin’s lymphoma—0.20%; non-Hodgkin’s lymphoma—0.49%; sarcoma—0.19%; and prostate cancer—0.57%. All of these cancer subtypes have been recognized by the American Academy of Sciences as being related to toxic chemicals/dioxins. Based on the results that liver cancer incidence in exposed veterans was 0.93%, the authors also proposed adding liver cancer to the list of cancers related to toxic chemicals/dioxins. For a better understanding of the effects of dioxins on cancer incidence in exposed veterans, we calculated the risk ratio (RR) for each type of cancer in the study based on the study data. The results are presented in Table 3 below.

The results of the risk ratio calculations showed that cancer incidence in exposed Vietnamese veterans is higher than that in unexposed groups, with a range that is 3.24 times (in respiratory cancer) to 12.8 times higher (in Hodgkin’s lymphoma). The risk ratio for all cancer subtypes was 3.8 (95% CI: 3.29–4.38, *p* < 0.0001).

Do et al. in 2009 [26] also conducted research on the relationship between Agent Orange and cancer rates in Vietnamese people 30 years after the war. The study used data on cancer status that were either self-reported by the respondents or provided by others obtained from the Vietnam National Health Survey (N = 158,019) combined with data on military herbicide exposure obtained from information on the military activities of the US and allied troops during the war. Self-reported and proxy-reported cancer status were collected from the 2001–2002 Vietnam National Health Survey (VNHS). A stratified random sample of 1200 communes (out of a total of 8926 communes) and 36,000 households was interviewed from November 2001 to November 2002. The VNHS gathers individual data on self and proxy-reported morbidity. More precisely, the survey instrument asks the main respondent about all household members’ health conditions. In addition to the VNHS, measurements of military herbicide exposure are constructed using the US Military Assistance Command Data Management Agency’s Herbicide Report System (HERBS) file. A geographic information system (GIS) was further developed to facilitate the estimation of exposure from HERBS file data. Records combine the Combat Activities file, the South-East Asia Database, and the Combat Naval Gunfire File (CONGA) and cover the combat activities of allied forces over the period between October 1965 and June 1975 with the exception of a few missing months. Additional information on agricultural herbicide use, cigarette and alcohol consumption, and coal and firewood expenditures were obtained from the 2004 Vietnam Household Living Standards Survey to look at household-level confounding factors. The multivariate estimation method was used to analyze the data.

The study results did not show a significant difference in cancer rates between the communes infected with the military herbicide and the uninfected communes. Meanwhile, when only focusing on communes with toxic contamination, they found a positive relationship between exposure and cancer rates reported in 2001–2002. The results suggest that among communes that were previously exposed, an increase of 10 percent in herbicide exposure is associated with a 2 percent increase in the probability of reporting suffering from cancer.

In 2021, Nguyen et al. [27] conducted a study on the health status of Agent Orange victims in some southern provinces of Vietnam with 1675 participants, including 1107 men and 568 women with an average age of 61.9 years, by means of physical examination combined with structured questionnaire. The results are summarized in Table 4.

However, there was no control group that was unexposed to dioxins in this study. Meanwhile, we could not compare the obtained cancer incidence with the national cancer rate because the study was carried out on a particular population (mainly the elderly with an average age of 61.9 years). Evaluating the risk ratio of cancer subtypes by using data from the study by Le et al. [25] with the incidence of the unexposed group (19,076 participants) also could not be conducted because of a lack of information on gender rate in the unexposed group.

## 4. Discussion

Epidemiological studies on the relationship between dioxins and cancer incidence in Vietnamese people are relatively few. Using a search strategy and selection criteria, we found only three studies. Based on the results, we have calculated the risk ratios and compared them to those of other studies in other countries around the world.

The results show that the risk ratios of all cancers and different cancer subtypes in Vietnamese people exposed to dioxins (with high levels in the contaminated areas of Vietnam caused by the US army during the war) were higher (3.76 times for all cancers; 5.22 times for soft tissue sarcoma; 11.33 times for Hodgkin’s; and 2.97 times for non-Hodgkin’s lymphomas) than those in other countries where the examined people were exposed to lower amounts of dioxins (mainly caused by occupational exposure or environmental pollution incidents) (Table 5). 

Despite an increased risk ratio for lung, prostate, and liver cancer in the dioxin-exposed Vietnamese people in our study, there is still only limited or suggestive evidence of an association between dioxins and these cancer subtypes in other studies. 

One new study of U.S. Vietnam veterans who attended a Veterans Affair (VA) Medical Center examined hepatocellular carcinoma comorbid with dual diagnoses of cirrhosis and hepatitis C virus and exposure to Agent Orange (determined by self-report) (Krishnamurthy et al., 2016) [28]. Although the estimated risk was elevated, no statistically significant association was found between exposure to Agent Orange and hepatocellular carcinoma. Such null findings are consistent with studies of other cohorts of U.S. Vietnam veterans as well as those from Australia and Korea. Although the study of Krishnamurthy et al. (2016) reported modest evidence of excess liver cancer among Vietnam veterans using VA Medical Center services, the weak design, nonspecific exposure, and confounding factors remain a concern for interpreting its results. The lack of evidence of an association between exposure and hepatobiliary cancers in the well-designed and exposure-characterized occupational studies does not support an association. Despite the evidence of TCDD activity as a hepatocarcinogen in animals, the evidence from epidemiologic studies remains inadequate to link dioxins with hepatobiliary cancers, which have a relatively low incidence in Western populations. Overall, the available evidence does not support an association between dioxins and hepatobiliary cancers.

Ovadia et al. (2015) [29] conducted an analysis of the relationship between self-reported Agent Orange exposure and long-term outcomes among prostate cancer patients. Data for this analysis were drawn from the Shared Equal Access Regional Cancer Hospital database of 1882 men undergoing radical prostatectomy for prostate cancer between 1988 and 2011 at six VA health care facilities; 333 men (17.7%) were considered to be Agent Orange-exposed. The clinical outcomes reported included the pathologic Gleason Score, pathologic stage, and postoperative pathologic and treatment characteristics as well as prostate cancer-specific death. Cox proportional hazards regression modeling was used to assess the relationship between exposure to Agent Orange and biochemical recurrence, secondary treatment, metastases, and prostate cancer-specific mortality. Models were adjusted for age, race, clinical stage, PSA level, BMI, center, and biopsy Gleason sum. Agent Orange exposure was not found to be associated with biochemical recurrence (HR = 1.21, 95% CI 0.99–1.49), secondary treatment (HR = 1.21, 95% CI 0.97–1.5), metastases (HR = 0.93, 95% CI 0.30–2.66), or prostate cancer-specific mortality (HR = 0.89, 95% CI 0.46–1.85). The study was generally well-conducted, with excellent clinical data and follow-up available within the VA system. Although Agent Orange exposure included an additional level of service location verification to self-report, this measure is still only a proxy for actual initial and subsequent exposure levels. However, a previous study of 93 men by Li et al. (2013) [30] showed good correlation between self-reported Agent Orange exposure and dioxin TEQ levels in adipose tissue. The study’s negative results may be applicable to the relationship between Agent Orange and prostate cancer progression but do not directly address initiation and incidence.

In contrast to the results of liver and prostate cancer epidemiological analysis, studies of Vietnam veterans are largely suggestive of modest associations between dioxins exposure and lung cancer incidence and mortality. In studies of U.S. veterans, a significantly increased risk of lung-cancer was found in Army Chemical Corps (ACC) veterans who used herbicides in Vietnam (Cypel and Kang, 2010) [31]), and an increased risk of lung cancer was associated with increased serum TCDD concentrations in Ranch Hand veterans (Pavuk et al., 2005) [8]. The Australian cohort studies of Vietnam veterans (ADVA, 2005) [32], which presumably cover a large portion of exposed soldiers, showed a higher than expected incidence of and mortality from lung cancer. The main limitations of the Australian and American ACC studies are that there was no assessment of exposure and that some potential confounding variables, notably smoking, could not be accounted for. Additionally, the Korean Veterans Health Study (Yi et al., 2014) [33] found modestly elevated, but not statistically significant, relative risks of both lung cancer incidence and mortality compared with the general population and high- versus low-exposure groups. The results were not adjusted for smoking, but earlier self-reported information from a large portion of the cohort indicated that smoking behavior did not appear to be related to the extent of a veteran’s exposure to herbicides. 

The limited evidence of an association between dioxins and cancer is most likely due to a lower amount of dioxins in these studied populations. The amount of dioxins in some hotspots of Vietnam is large enough to increase their concentration in the blood of exposed people, leading to markedly increased risk ratios. This finding corresponds with the conclusion from the study conducted by Xu et al. [14], which showed that the morbidity and mortality of some types of cancers were related to an increased dioxin concentration in the environment and in the patient’s blood. Therefore, it is important to analyze dioxin levels in the contaminated environment and in biological organisms when studying cancer incidence in a population exposed to dioxin.

We planned to conduct a meta-analysis from epidemiological dioxin studies in Vietnamese people but could not complete one because of the limited and heterogeneous data. There was no information about gender or other cofactors in the study by Le et al. [25], which may influence the reliability of the study’s results. Therefore, the study cannot be used for further meta-analysis. Nguyen et al. [27] conducted an epidemiological study with a small number of participants (n = 1675), which may have made their results less representative. The study by Do et al. [26] was based on questionnaire data and that lacks information about objective assessment (blood and tissue levels of dioxin). These limitations may reduce the reliability of the obtained results, and further studies are needed to resolve these problems.

## 5. Conclusions

Based on the literature review of the relationship between dioxins and cancer incidence, the following conclusions can be proposed:

1. The results of epidemiological studies confirm the association between exposure to dioxins and the development of cancer subtypes (soft tissue sarcoma; Hodgkin’s and non-Hodgkin’s lymphoma; and lung, prostate, and liver cancer) in some contaminated areas of Vietnam.
2. The incidence of these cancer subtypes in Vietnam veterans affected by Agent Orange is much higher than the results of studies published in other parts of the world because of the high levels of dioxins in South Vietnam, which is where Agent Orange was sprayed during the war.
3. In the future, epidemiological studies on the relationship between dioxins and cancers in Vietnam should be conducted using improved research methods to correct the methodological problems of previous Vietnamese studies. In addition, the reliability of the research results needs to be achieved by assessing TCDD exposure level with a large number of study participants.

## Figures and Tables

**Table 1:** Characteristics and results (RR) of some of the epidemiological studies on relationships between dioxins and human cancer development. Reproduced from Xu et al. [14], published by *Sci. Rep*., 2016.

No.	Study	Country/Cohort	Time Period	Exposure Way	Exposure Assessment	Cancer Types	Gender	No. of Cancer Cases/Cohort or Controls	Age (Years)	RR
	External exposure to TCDD and cancer incidence (exposure incidence)									
1	Kogevinas [15]	Part of IARC	1955–1988	Occupational	Job and company records, questionnaires	All cancers, breast cancer	F	29/701	N/A	2.22
2	Read [16]	New Zealand	1970–2001	Nonoccupational	Individual records	All cancers	F/M	8013/375,583	N/A	0.91–1.11
3	Pesatori [3]	Italy, Seveso	1977–1996	Industrial accident	Measurements of TCDD soil levels	All cancers	F/M	2122/218,761	0–74	Zone A: 1.03 Zone B: 1.00 Zone R: 0.96
	Blood level of TCDD and cancer incidence (blood incidence)									
4	Pavuk [8]	USA, Vietnam Veterans	1982–2003	Vietnam war	Physical examinations and blood samples	All cancers	M	402/1482	Mean 63.7	1.6
5	Warner [12]	Italy, Seveso, SWHS cohort	I:1976–1996, II:1997–2009	Industrial accidents	Interviews, physical and blood samples, and sample examination	All cancers, breast cancer	F	66/981	0–40	2.77
6	Ott [11]	Germany, Ludwigshafe	1959–1992	Occupational	Questionnaires and blood samples	All cancers	M	31/243	N/A	1.3

**Table 2:** Analysis of the association between external exposure to TCDD and cancer incidence. Reproduced from Xu et al. [14], published by *Sci. Rep*., 2016.

Type of Cancer	Pooled Risk Ratio (RR)	95% CI
All cancers	1.01	0.97–1.06
Soft tissue sarcoma	1.37	0.97–1.93
Hodgkin’s Lymphoma	1.13	0.83–1.54
Non-Hodgkin’s Lymphoma	1.09	0.92–1.30

**Table 3:** Risk ratio (RR) of cancer among Vietnamese veterans exposed to dioxins in the years 1962–1975.

	Exposed Group		Unexposed Group		Risk Ratio	
	*N* = 28,817		*N* = 19,076			
Diseases	Number	Incidence (%)	Number	Incidence (%)	RR	95% CI
All cancers	1262	4.38	220	1.15	3.80	3.29–4.38
Soft tissue sarcoma	54	0.19	5	0.03	7.15	2.86–17.87
Hodgkin’s Lymphoma	58	0.20	3	0.02	12.80	4.01–40.83
Non-Hodgkin’s Lymphoma	142	0.49	29	0.15	3.24	2.17–4.83
Carcinomas of the lung, bronchus, and trachea.	577	2.00	111	0.58	3.44	2.81–4.21
Prostate cancer	164	0.57	37	0.19	2.93	2.05–4.19
Liver cancer	267	0.93	35	0.18	5.05	3.55–7.18

Note: Data from Le et al. (2007) [25].

**Table 4:** Numbers and incidence of cancer among residents exposed to Agent Orange living in some of the provinces of South Vietnam.

Type of Cancer	Number	Incidence (%)
All cancers	163	9.73
Lung cancer	17	1.01
Liver cancer	23	1.37
Kidney cancer	34	2.03
Prostate cancer	69	6.23
Ovary cancer	7	1.23
Uterine cancer	13	2.29

Note: Data from Nguyen et al. (2021) [27] with 1675 participants including 1107 men and 568 women.

**Table 5:** Comparison of cancer incidence risk ratio for Vietnamese people with people from other countries exposed to dioxins.

Type of Cancer	Risk Ratio in Dioxin-Exposed Vietnamese in the Study of Le et al. *	Risk Ratio in Dioxin-Exposed Patient from other Countries **	RR_Vietnam_ /RR_Other Countries_
All cancers	3.80	1.01	3.76
Soft tissue sarcoma	7.15	1.37	5.22
Hodgkin’s Lymphoma	12.80	1.13	11.33
Non- Hodgkin’s Lymphoma	3.24	1.09	2.97
Lung cancer	3.44 (4.40)	No data	-
Prostate cancer	2.93 (21.24)	No data	-
Liver cancer	5.05 (7.48)	No data	-

Note: Data (*) from study of Le et al. (2007) [25], data in parentheses from Nguyen et al. (2021) [27], and (**) from Xu et al. (2016) [14].

## Data Availability

Data supporting reported results can be found in Proceeding of International Conference Protocol on 19 December 2021, published by Vietnam Association for Victims of Agent Orange/dioxin—VAVA.
